# User Requirements of the Integrated Home-Based Rehabilitation Tool, Neurorehabilitation Ecosystem for Sustained Therapy: Multicenter Focus Group Study With Stroke Survivors, Caregivers, and Health Care Professionals

**DOI:** 10.2196/79382

**Published:** 2026-04-29

**Authors:** Merle Matijsen, Cheriel Hofstad, Susana Rodriguez Gonzalez, Xavier Buxó, Vera Stara, Sara Leonzi, Margherita Rampioni, Anna Mura, Noël Keijsers

**Affiliations:** 1 Department of Research, Sint Maartenskliniek, Hengstdal 3, Nijmegen, 6500 GM, The Netherlands; 2 Department of Sensorimotor Neuroscience, Donders Institute for Brain, Cognition and Behavior, Radboud University Nijmegen, Nijmegen, The Netherlands; 3 Department of Physical Medicine and Rehabilitation (PMR), Neurorehabilitation Unit, Vall d'Hebron Hospital Universitari, Barcelona, Spain; 4 PMR Research Group, Vall d'Hebron Institut de Recerca, Barcelona, Spain; 5 Centre for Innovative Models for Aging Care and Technology, National Institute of Health and Science on Aging, IRCCS INRCA, Ancona, Italy; 6 Institute of Neurosciences, University Miguel Hernández-CSIC, Alicante, Spain; 7 Department of Sensorimotor Neuroscience, Donders Institute for Brain, Cognition and Behavior, Radboud University Medical Center, Nijmegen, The Netherlands

**Keywords:** home–based rehabilitation, user requirements, qualitative research, stroke, healthcare professionals, multi–country

## Abstract

**Background:**

Due to the high burden on health care, home-based rehabilitation (HBR) has gained increasing interest. A new HBR program for stroke survivors, containing a gaming app with upper-limb exercises, monitoring system, and virtual coach was being developed.

**Objective:**

This study aimed to assess the user requirements of an HBR tool including a gaming app, monitoring system, and virtual coach, and to examine potential differences between end users and countries.

**Methods:**

Thirteen stroke survivors, 12 caregivers, and 15 health care professionals from centers in the Netherlands, Italy, and Spain, participated in focus groups or interviews. Each center used the same interview guide with open questions about each component. An inductive thematic analysis was conducted separately at each center, and results were combined during a physical meeting.

**Results:**

User requirements were categorized into three main themes: (1) customization: aligning with individual preferences and capabilities; (2) motivational elements: these included reminders, a variety of levels and games, and ease of use; and (3) feedback elements: maintaining interactions with therapists. These themes apply to both home-based exercises as well as daily-life activities during HBR. There were minor differences between end users or centers.

**Conclusions:**

All end users across the participating countries emphasized the importance of integrating gamified exercises, monitoring, and virtual coaching into an HBR system. The user requirements for such a system can be categorized into three key areas: customization, motivational elements, and feedback elements.

## Introduction

The economic burden of stroke on health care systems is substantial due to large numbers of people affected by stroke and the high costs associated with acute treatment and long-term care. This treatment is necessary due to the diverse range of poststroke impairments, including reduced upper-limb function [1]. In 2019, over 12.2 million new stroke cases were reported worldwide, marking a 70% increase in stroke incidence since 1990 [2,3]. Due to population aging, this upward trend is expected to continue in the coming years [4], leading to even higher costs [5]. Furthermore, a shortage of medical personnel can arise due to the expected increase in stroke cases. To reduce costs and personnel, a shift in focus toward home-based rehabilitation (HBR) has recently emerged [6]. HBR offers additional benefits including reduced travel time for stroke survivors, the possibility to increase therapy intensity, increased accessibility allowing users to perform the activities throughout the day, and longer therapy duration [7].

Since higher training intensity leads to greater improvements in most patients [8,9], a critical element for the success of HBR is adherence. However, it is challenging with HBR to achieve adherence consistently, especially given that in general, treatment adherence tends to decrease over extended periods [10]. This decrease is often the result of low acceptability and usability, the inability to stay engaged, or integrate the practice into daily life [10]. Nonetheless, adherence to treatment is crucial for patients with chronic stroke, since behavioral changes in arm use require a substantial training dosage schedule [11]. Adherence rates in HBR for stroke vary significantly, ranging between 13% and 140% [10]. Several factors positively influence the adherence rate in HBR, including rehabilitation programs with game-like training exercises with multiplayer modes, sufficient variability in games, and adjustable difficulty levels. Moreover, the availability of monitoring and feedback options to track users’ progress and motivate users also increases adherence [10,12]. Technical support for issues, such as malfunction of the system, and the possibility to customize the system to meet individual users’ needs are also important considerations [10,12]. Implementing these factors in HBR can increase adherence and thus improve its overall success.

When considering HBR, multiple end users can be identified, such as stroke survivors, health care professionals, and caregivers. All these end users have a different relation to individuals with stroke. These different relations could result in different perspectives; for example, a health care professional might be more objective, whereas the caregivers and stroke survivors are more subjective. However, Demir et al [13] found that stroke survivors, health care professionals, and caregivers generally share the same priorities during stroke rehabilitation, focusing on improving the health condition post stroke, arm and leg mobility, and increasing independence. Nonetheless, HBR might require other demands compared to usual care. In home settings, caregivers and stroke survivors might prioritize ease of use, whereas health care professionals might focus primarily on health outcomes. As a result, health care professionals might have specific requirements for outcome visualization and monitoring, whereas caregivers are more concerned with the practical challenges of implementing rehabilitation therapy in a home setting. These potential differences underscore the importance of a flexible and adaptive system design that meets the needs of diverse users and should be carefully considered in developing a successful HBR system. However, the different viewpoints of these end users on user requirements for HBR is still unknown.

One advantage of HBR is its potential for scalability and implementation across multiple countries, making it accessible to a diverse range of populations. However, differences in health care systems and cultural practices across Europe must be considered. Across Europe, there are several clusters with comparable health care systems, with Spain, Italy, and the Netherlands belonging to the same cluster [14]. However, Italy and Spain present a higher overlap with each other compared to the Netherlands [14]. Additionally, health care systems in Italy and the Netherlands rely more on inpatient care, while Spain has a stronger outpatient care tradition compared to Italy and the Netherlands [15], suggesting that Spain may have greater experience with home-based exercises. There are also some cultural differences. For example, Italy and Spain are more collectivistic, while the Netherlands is more individualistic [16]. Thus, the Netherlands might be more receptive to HBR, since this is a more individualistic therapy form. Studies have even shown that countries across Europe use different cognitive emotion regulation strategies [17]. These differences, both in health care systems and cultural practices, can potentially influence the requirements of an HBR tool. To accommodate these differences in system design, a more prominent role for the coaches in countries less familiar with HBR and enhance group interaction features where social engagement is valued could be integrated. However, there is currently no information available on these possible differences.

Recently, within an international project involving partners from Italy, Spain, and the Netherlands, a new HBR solution was defined, called the Neurorehabilitation Ecosystem for Sustained Therapy (NEST). As of January 2021, this solution is still in the design phase and has not been developed. This innovative rehabilitation system offers an integrated rehabilitation solution for stroke survivors, featuring the following components: (1) the RGS (Rehabilitation Gaming System) app for smartphones, which offers gamified exercises using augmented reality for upper-limb training, focusing on finger, wrist, elbow and shoulder movements while holding a smartphone; (2) the RGS-wear, a smartwatch-based system for monitoring upper-limb movements during daily activities; and (3) a virtual coach, the agent for well-being assistance (AWA), integrated into both the RGS app and RGS-wear, providing motivational feedback on training performance and daily arm use as well as assistance in the use of the gamified exercises. The combination of these three components, which were found relevant in previous literature [10,18,19], makes NEST a unique HBR tool. However, whether specific user requirements for NEST exist and if the system fulfills these needs remains unclear.

In summary, no specific user requirements are known for a NEST-based HBR system, including optimization of gamified training exercises, monitoring of arm use, and virtual coaching. More insight into user requirements specific to NEST could help to further optimize and develop NEST. In addition, there is a lack of knowledge about its implementation across different end users and multiple countries. It is essential to conduct research with multiple end users and across different countries to gain more insight into user requirements. Therefore, the first aim of this study was to define the user requirements for a newly developed upper-limb training system that includes the three aspects of NEST for the end users: stroke survivors, health care professionals, and caregivers. Our second aim was to identify possible differences in user requirements among these end user groups and across three European countries (The Netherlands, Italy, and Spain).

## Methods

### Research Context

The NEST project is a collaborative effort between hospitals and industry partners aimed at developing effective rehabilitation technologies to enhance the recovery of function in patients with stroke. The industrial partners, Eodyne en Hankamp Rehab, are specialized in the development and exploitation of rehabilitation technologies. The clinical partners include Sint Maartenskliniek in the Netherlands, Vall d’Hebron University Hospital in Spain, and Instituto Nazionale di Ricovero e Cura per Anziani in Italy. All clinical centers have significant experience in stroke rehabilitation.

### Study Design

**Figure 1.**
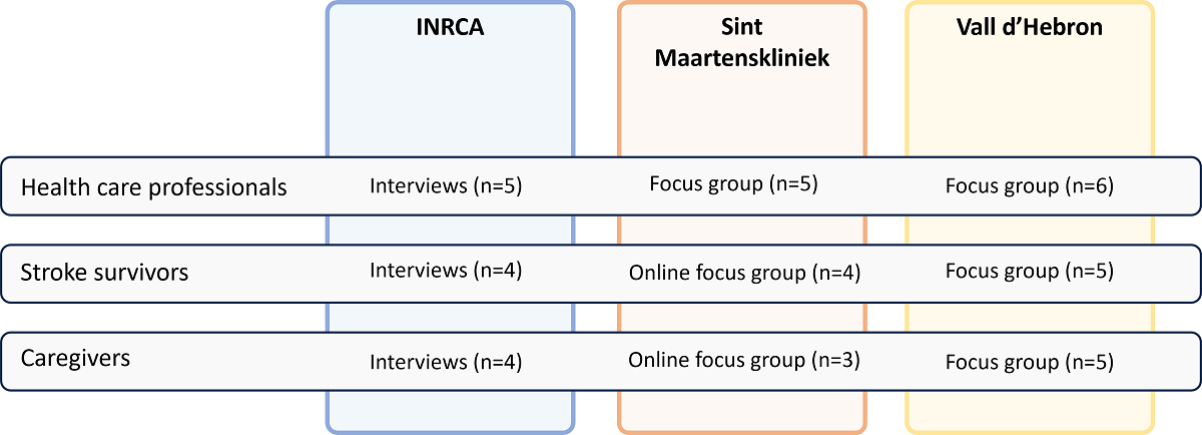
Overview of the focus groups, type of focus group and interviews in each center with the number of participants. INRCA: Instituto Nazionale di Ricovero e Cura per Anziani.

A multicenter, qualitative study was conducted at the three clinical sites planned to include three on-site focus groups, segregated by end user. The participants presented the three primary users of the NEST solution: (1) health care professionals, (2) caregivers, and (3) stroke survivors. The end user groups were identified based on their likelihood of being future system users. The planned group size for each group was 5 participants. However, recruiting took place in the beginning of 2022; therefore, difficulties arose due to COVID-19 restrictions. In addition, not all the clinical sites could perform on-site focus groups because of COVID-19 limitations. The Dutch partner performed online focus group sessions with caregivers and stroke survivors and an on-site focus group session with health care professionals. At the Italian clinical rehabilitation center, focus groups were not feasible; hence, all participants were individually interviewed. The Spanish partner conducted all the focus groups on site. An overview of the study design can be found in Figure 1.

### Ethical Considerations

All participants gave written consent before the start of the focus group or interview. Medical–ethical approval for this study was not required in Spain and Italy. In the Netherlands, the focus group protocol was submitted to Commissie Mensgebonden Onderzoek Arnhem/Nijmegen, which determined that a full ethical review was not required and granted approval to conduct the study (2022‐13564). In addition, this study was conducted in accordance with the Declaration of Helsinki of the World Medical Association.

### Participants

#### Health Care Professionals

Health care professionals, such as physiatrists, physical therapists, or occupational therapists, with a minimum experience of 5 years in the rehabilitation of stroke survivors were included.

#### Stroke Survivors

Potential stroke survivors were identified and contacted by clinicians at the three centers using electronic medical records. Interested participants were then assessed for eligibility based on inclusion and exclusion criteria.

The inclusion criterion was being an adult stroke survivors (18 years or older) with a paretic arm. Exclusion criteria were individuals with aphasia and/or cognitive impairment (Mini Mental Examination score of <21), those with minimal smartphone proficiency, and those affected by other neurological disorders.

#### Caregivers

Informal caregivers were recruited via the stroke survivors who participated in the study. In addition, social media, such as Facebook, was used to recruit caregivers. Across all three countries, informal caregivers were consistently defined as individuals who provide unpaid assistance and support to stroke survivors who cannot fully care for themselves independently. Caregivers could participate if they had been or currently were taking care of a stroke survivor with a poststroke impaired arm. They were excluded if they had minimal smartphone proficiency. If caregivers showed interest, they were further assessed to determine if they met the inclusion and exclusion criteria.

### Structure of the Focus Groups and Data Collection

Focus groups and interviews were conducted by a native language–speaking interviewer accompanied by an assistant who took notes. Both were experienced researchers at the respective centers. Each focus group lasted between 1 and 2 hours, with breaks available for stroke survivors if needed. Interviews lasted approximately 30 minutes. All three clinical centers used the same English interview guide, which was translated into the local language for consistency across focus groups and interviews. Questions were tailored to the perspective of the participant group. The detailed English interview guide for stroke survivors can be found in Multimedia Appendix 1, with additional details about the interview guide for caregivers and health care professionals. The interviewer started with an introduction of the NEST system, including its three components (the RGS app, RGS-wear, and the AWA coach), followed by brief videos illustrating how NEST operates. In addition, the researcher explained that the videos showed a prototype version of NEST and that the final components were not physically available yet. They also mentioned that the user requirements obtained with the study are necessary for further advanced development of NEST. Participants were then asked for permission to audio record the session. Participants were questioned on three central topics: HBR in general, participants’ perspective on the three components of NEST, and their general opinion about NEST. While user requirements for system design were explored, the focus groups also allowed discussion of participants’ preferences, expectations, and general opinions. At the start of each topic, the interviewer posed an open question, for example, “What do you need in order to be able to use the RGS–mobile app in daily practice?” to initiate an open discussion. To stimulate further discussion, more specific questions were asked, such as, “What do you think of the different exercises and games?” If both the participants and researcher felt all points were discussed, the next topic was introduced. After the focus group or interview, participants received a summary to check if they had any additional remarks.

### Data Analysis

Each clinical site independently transcribed all recorded audio tapes of the interviews and focus groups in full. Manual transcription was conducted at each site, and analysis was performed using Atlas.ti software (Lumivero). An inductive content analysis approach was carried out, in which an analytical framework is created in multiple steps: familiarization, coding, identifying themes, reviewing themes, and defining themes [20,21]. First, each transcript was read in full by the researcher to become familiar with the content. Second, the researcher assigned codes that closely reflected the participants’ wording, called “open coding” [22]. Third, the initial codes were refined and organized into categories (eg, multiplayer mode and technical skills) [23]. Lastly, two researchers at each site discussed categories to create concept and initial themes until consensus on the themes was reached. In instances where consensus was not achieved, a third researcher was consulted to make the final decision.

After the three separate inductive thematic analyses at each clinical center, two researchers from the Spanish site, one from the Dutch site, and two from the Italian site met in person to integrate their findings. The objective of this meeting was to identify user requirements across sites. The method of Van der Elst et al [24] was used for combining results. First, each site summarized and presented their site-specific factors to the others. Clarifying questions were asked and additional contextual information was provided where needed to ensure a shared understanding of the findings. Second, all sites prepared sticky notes with their analytic categories, each site using a different color. During an in-person collaborative mapping meeting, comparable categories from different sites were grouped to identify similarities and differences, forming the initial synthesis of site-level findings into broader cross-site patterns. Third, all researchers engaged in a group discussion to interpret the similarities and to develop higher-order themes reflecting shared user requirements. Differences between sites were discussed explicitly and examined in relation to contextual factors, rather than being treated as discrepancies to be resolved. Finally, themes were refined and prioritized based on their relevance to the study objectives and their significance across sites. When divergent interpretations occurred, these were retained as country-specific findings.

The sticky notes also contained information on which end user group discussed each category, allowing for the identification of similarities and differences between end user groups. All steps were repeated for each topic: general HBR, the RGS app, RGS-wear, and the AWA coach. Lastly, all factors were categorized into essential requirements, desirable features, and contextual factors by the first author; this classification was reviewed and approved by the other authors.

## Results

### Overview

In total 13 stroke survivors, 12 caregivers, and 15 health care professionals participated in the study. Participants were distributed into six focus groups and 13 interviews. Basic characteristics of the participants are shown in Table 1. There are no differences in characteristics between the participants of the three clinical sites, except for a longer time since stroke in stroke survivors at the Dutch site.

An overview of the essential requirements, desirable features and contextual factors can be found in Table 2. All participants expressed enthusiasm for NEST, highlighting the relevance of the three components, and indicated a potential interest in testing it.

**Table 1.** Characteristics of participants in the focus groups and interviews.

	INRCA			VH			SM		
	SS^a^	CG^b^	HP^c^	SS	CG	HP	SS	CG	HP
Participants, n	4	4	5	5	5	5	4	3	5
Sex (male), n	2	1	1	4	1	2	4	1	2
Age (years), median (range)	67 (61-71)	53 (45-68)	35 (31-55)	63 (58-67)	47 (25-63)	43 (28-56)	57 (44-58)	61 (29-80)	45 (35-60)
Time since stroke (months), median (range)	2 (1-3)	2 (1-3)^a^	—^d^	3 (1-5)	3 (1-5)^a^	—	72 (8-173)	6 (5-78)^a^	—
Experience (years), median (range)	—	—	10 (4-30)	—	—	8 (3-29)	—	—	19 (8-28)

a SS: stroke survivor.

b CG: caregiver.

c HP: health care professional.

d Not applicable.

**Table 2.** Essential requirements, desirable requirements, and contextual factors of NEST^a^.

Components	Factors
Essential requirements	
RGS^b^ app	Variety of levels and games
RGS app and RGS-wear	Simple and intuitive Technical support Privacy concerns RGS app without RGS-wear Initial training
* *RGS-wear and AWA^c^ coach	Quality of movements
AWA coach	Goal-oriented coach Positive attitude Reminders^d^ Feedback^e^
Desirable features	
RGS app	Multiplayer mode Adding more functionalities Adjust to preferences
RGS-wear	Circumstance stroke survivor
AWA coach	Feedback by stroke survivor Emotional slider Frequency reminders Type of signal
Contextual factors	
General	Additional to on-site therapy Timing home-based training Role caregiver^f^
RGS app	Level of lesion
RGS app and RGS-wear	Monitoring^g^ Basic technical skills
RGS-wear	Self-responsibility

a NEST: Neurorehabilitation Ecosystem for Sustained Therapy.

b RGS: Rehabilitation Gaming System.

c AWA: agent for well-being assistance.

d On both games and daily tasks.

e During and after gameplay and from the virtual coach and therapist.

f Either supportive or limited.

g Involving the (local) therapist.

### Similarities in User Requirements Across End User Groups and Countries

#### The RGS App

Several factors for the RGS app were collectively identified by the three clinical sites, categorized into four main themes: game requirements, technical requirements, role of the therapist, and customization. Game requirements included a need for a variety of gamified exercises and difficulty levels within games. A multiplayer mode was also discussed, which could potentially boost motivation but might also lead to demotivation. To prevent unfair competition resulting from differences in upper-limb function, customization is essential in multiplayer mode, which should be designed to ensure fairness and minimize user burden. This concern is shown in the following quote:

*A collaboration is bothersome if something is dependent on you … how would that work? If you do something together and you fail, and the whole team fails. I would not like that.*

[Stroke survivor, the Netherlands]

The technical requirements underscored that the RGS app must be simple and intuitive to use in order for them to stay motivated. However, although a basic level of technical skills is still necessary for stroke survivors. Additionally, technical support must be available to address any issues that arise. The importance of the therapist’s role during the use of NEST was emphasized. The therapist should provide clear instructions before patients take the RGS app home. Initial sessions in the outpatient clinic introducing the system were suggested so that the therapist could define both the goals and the treatment pathway best suited to the patient. In addition, NEST should provide continuous supervision and monitoring during use. In this way, the therapist would ensure support, motivation, and movement corrections. Such monitoring is particularly important in the early stages of HBR and may gradually decrease over time. The following was said during an interview:

*The therapist should motivate the patients and control that the exercises are done in the most appropriate way.*

[Stroke survivor, Italy]

The factor customization is related to the personalization of the RGS app to suit individual stroke survivors. Depending on the location of the lesion and capabilities of the stroke survivor, specific games should be selectable or not. Furthermore, the ability to adjust games to personal preferences, such as displaying the digital hand on the paretic side, modifying difficulty levels, and tailoring games to the users’ hobby was highlighted. Also, the possibility to add more functionalities, such as balance games, were mentioned.

#### RGS-Wear

The factors concerning RGS-wear were categorized into three main topics: monitor requirements, technical requirements, and customization. With monitoring, the smartwatch should be capable of measuring both the quality and quantity of movements to gain insight into whether compensation strategies were used during both games and daily life. For example, if a stroke survivor focuses on reaching movements, the RGS-wear should be able to register a reaching movement. These measurements should be saved and analyzed periodically by the therapist. Analysis of arm use in daily life also enables the therapist to further specify and tailor interventions, which was also described by a therapist:

*Upper limb movement restoration during stroke recovery is a particularly important pillar of rehabilitation practice.*

[Health care professional, Spain]

The technical requirements of RGS-wear align with those of the RGS app: it needs to be simple and intuitive to use, although a basic level of technical skills is still necessary. Participants indicated that if the smartwatch did not function properly, it would lead to demotivation in using the system. Additionally, a clear instruction on how to use the smartwatch must be provided. The desire to use the RGS app independently of RGS-wear was also discussed.

*It needs to be user friendly, I think. Especially since people with a stroke are often a bit older, because the smartwatches I know are for example quite small.*

[Caregiver, the Netherlands]

Customization options for RGS-wear should take into consideration the user’s specific circumstances, such as use of a sling and esthetics. Esthetics refer to aspects such as the size, design, and color of the smartwatch. Some participants, for example, preferred smaller, more traditional designs over large, modern-looking smartwatches.

#### AWA Coach

For the AWA coach, the factors were categorized into three main themes: reminder requirements, feedback requirements, and customization*.* Reminders should not only prompt the stroke survivor to start the games but also encourage the use of the paretic arm during daily tasks. These reminders should be presented with a positive attitude. The importance of reminders was highlighted:

*Coach reminders could become an important part of the rehabilitation in some of the post–stroke patients in order to improve their motivation and adherence to treatment. These reminders should also be optional and tailored to user’s specific needs.*

[Health care professional, Spain]

No specific interest in an avatar was expressed across end user groups. Feedback on upper-limb performance should be provided during gameplay, after gameplay, and during daily activities for which RGS-wear data can be used. Personalized feedback from the therapist would also be very helpful and motivating. In addition, also some patients would like to send feedback to the AWA coach in case of necessity. For example, a health care professional mentioned:

*Games offered in home rehabilitation should include a feedback system for the patient to check his progress (eg, game score, growing tree, graph etc). There should be weekly feedback showing daily activities done, use of the arm, etc. A notification should be sent to the therapist if the patient is playing or exercising less than expected.*

[Health care professional, Italy]

The frequency of reminders and feedback on daily life use of the paretic arm should be tailored to each user’s preferences. Cognitive ability could play a role, since some participants may simply forget to perform the exercises, making reminders more important. Feedback should be goal-oriented and therefore tailored to the user. The type of signal for reminders should be customized to the stroke survivor. However, no definitive consensus on the preferred signal type emerged during the focus groups and interviews. In general, verbal and written reminders seemed to be fine.

### Differences Between End User Groups

An overview of similarities and differences across end user groups and countries for each topic is shown in Table 3. Overall, most user requirements focused on the needs of stroke survivors. Also, caregivers and health care professionals primarily considered requirements from the perspective of stroke survivors, placing less emphasis on their own requirements for system design. Overall, the end user groups were largely in consensus regarding the user requirements of NEST. However, a few topics were not addressed by all groups, with caregivers not addressing all topics mentioned by stroke survivors and health care professionals. First, caregivers did not share opinions about the early introduction of the system, possibly already in the clinic. They also did not address the ability of the smartwatch to measure the quality of movements. Lastly, caregivers did not express a preference for goal-oriented feedback and the method of delivering feedback. In contrast, caregivers showed interest in having access to the data collected by RGS-wear, a point not mentioned by stroke survivors and health care professionals. Stroke survivors mentioned the possible negative impact of caregivers on motivation if caregivers push the stroke survivors too much at home. Therapists focused on the upper-limb function of stroke survivors and which patients should be able to use the system.

**Table 3.** Overview of similar and different factors among the 3 sites. The dashed line means that the topic was mentioned by health care professionals and stroke survivors. The solid line means that the topic was only mentioned by health care professionals. The absence of a line means that the topic was mentioned by all end users and therefore there was consensus.

Topics			Italy	Netherlands	Spain
General home–based					
	Integrating requirements				
		HBR^a^ as additional intervention	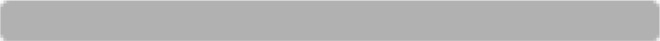		
		Getting familiar	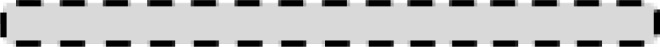		
		Monitoring by therapist	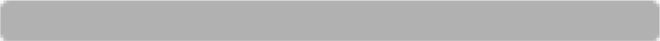		
		Involve local therapist		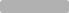	
	Motivational requirements				
		Motivational components	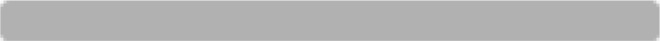		
		Timing home–based training	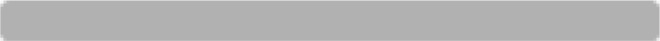		
		Supportive caregiver	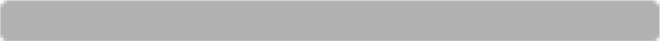		
		Limited role caregiver		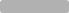	
RGS^b^ app					
	Game requirements				
		Variety of levels and games	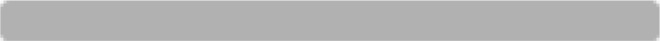		
		Multiplayer mode	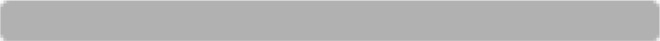		
		Adding more functionalities			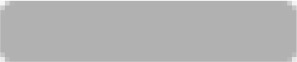
	Technical requirements				
		Simple and intuitive	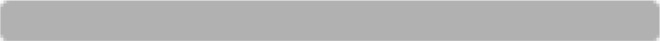		
		Basic technical skills	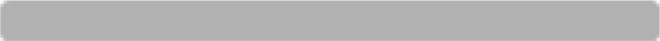		
		Technical support	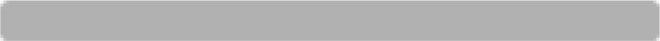		
		Privacy concerns			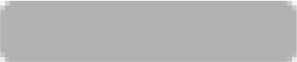
	Role of the therapist				
		Supervision and monitoring	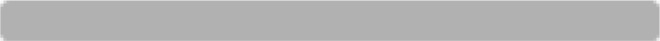		
		Initial sessions	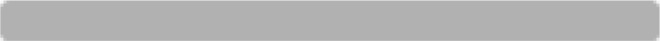		
	Customization				
		Adept to level of lesion	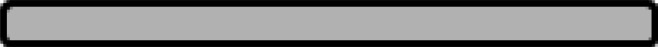		
		Adjust to preferences	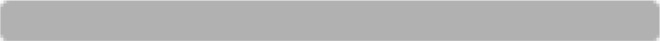		
RGS-wear					
	Monitor requirements				
		Quality of movements	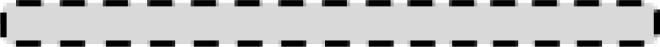		
		Analyzed by therapist	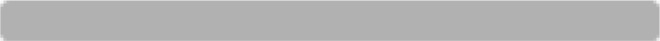		
		Self–responsibility stroke survivor	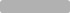		
	Technical requirements				
		Simple and intuitive	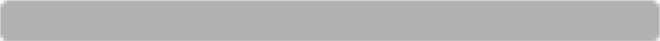		
		Basic technical skills	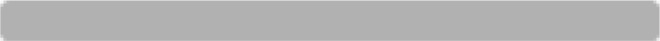		
		Clear instructions	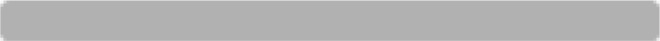		
		Privacy concerns		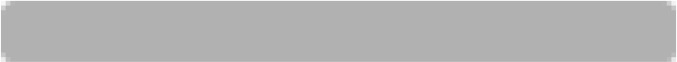	
	Customization				
		RGS app without RGS–wear	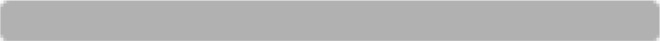		
		Circumstances stroke survivor	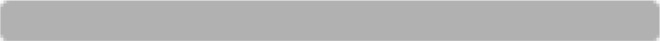		
AWA^c^ coach					
	Reminder requirements				
		Positive attitude	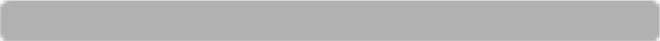		
		Reminder for doing games	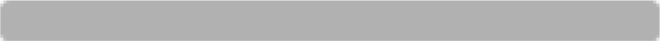		
		Reminder for daily tasks	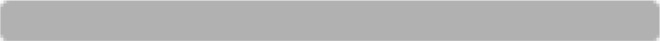		
		No interest in avatar	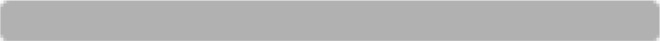		
	Feedback requirements				
		Feedback during game	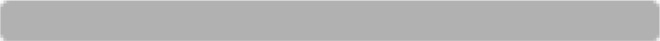		
		Feedback after game	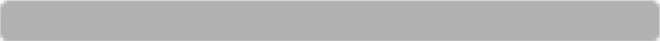		
		Feedback during daily tasks	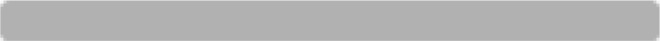		
		Feedback from therapist	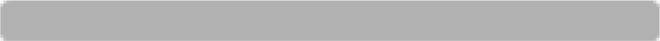		
		Send own feedback to coach		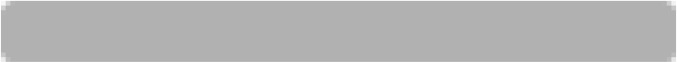	
		Emotional slider			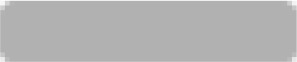
	Customization				
		Frequency reminders	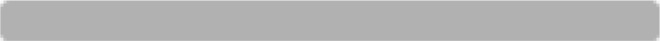		
		Type of signal	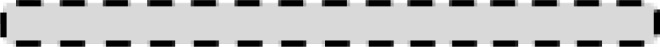		
		Goal–oriented coach		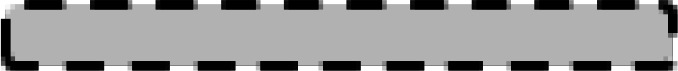	

a HBR: home-based rehabilitation.

b RGS: Rehabilitation Gaming System.

c AWA: agent for well-being assistance.

Although caregivers and health care professionals primarily prioritized the needs of stroke survivors, certain user-specific requirements emerged. Caregivers emphasized that NEST should function without their help, as they should not be responsible for monitoring or motivating its use. Given their caregiving responsibilities, additional tasks could increase their burden and shift their role from a supportive partner to that of a therapist or even nurser. Instead, they preferred the option to track progress when interested or when they considered it was necessary to intervene. Additionally, caregivers expressed interest in playing games together with stroke survivors, enabling active participation rather than solely in a motivational capacity role.

Health care professionals considered whether NEST would reduce or potentially increase their workload. They noted that stroke survivors often require assistance with exercises, and if therapists still need to provide coaching and feedback on their training, their burden could even rise. Collaborative management of NEST with local therapists could reduce the burden. However, they emphasized that NEST could support stroke survivors’ vitality, which remains the most important. Additionally, therapists highlighted that smartwatch data could offer valuable insights into motivation strategies, which they can use during on-site therapy, resulting in increased quality of the therapy.

### Differences Between Countries

All countries showed high overlap in topics discussed. Some differences between sites were observed, which may be related to differences in patient characteristics across countries, such as the higher proportion of patients with chronic stroke at the Dutch site. However, a few differences are worth noting. At the Dutch site, therapists and stroke survivors highlighted the beneficial effect of involving local therapists in NEST. Additionally, Dutch stroke survivors mentioned that caregivers could negatively impact motivation due to the already high burden of caregiving tasks. Privacy concerns regarding the use of the RGS app and smartwatch were raised both at the Spanish and Dutch sites. Both sites also mentioned the importance of making the AWA coach goal-oriented and enabling stroke survivors to provide feedback on their own performance. The Italian site shared similar views, highlighting the importance of awareness and self-responsibility among stroke survivors. They noted that the smartwatch’s tracking of daily arm use could increase the stroke survivors’ awareness of their functionality, thereby empowering them. Unique to the Spanish site was the suggestion to expand the RGS app to include mobility and balance exercises.

### General Feedback on HBR

In addition to the specific requirements for NEST, this study identified broader user requirements for HBR. The factors discussed about general HBR were categorized into two main themes: integration requirements and motivational requirements*.* Integration requirements refer to incorporating HBR into the overall rehabilitation program as an additional intervention rather than a replacement for on-site rehabilitation. Key aspects include providing adequate instructions for using the HBR system, getting familiar with the HBR system during rehabilitation before starting the intervention at home, and ensuring ongoing monitoring by the therapist during home use. In addition, the timing for initiating the HBR training period should be personalized for each stroke survivor. The motivational requirements emphasize the importance of incorporating elements that enhance motivation with the HBR system. The caregiver must have a supportive role throughout the training period, helping to sustain engagement and adherence to the program.

## Discussion

The first objective of this study was to identify the user’s needs of a newly developed upper-limb training system that includes gamified exercises, monitoring, and a virtual coach. This was investigated with focus groups and interviews with stroke survivors, caregivers, and health care professionals in clinical centers in Italy, Spain, and the Netherlands. Participants across all user groups and countries highlighted the relevance of each component of NEST, the RGS app, RGS-wear, and the AWA coach. The participants recognized the potential of NEST, including these elements, as an HBR tool. In order for this combination to be successful, motivational elements such as gaming, feedback, and reminders are important. In addition, during the focus group sessions and interviews, several improvements were suggested, mainly concerning adding motivational components and enhancing system customization. If these suggestions are integrated, the system has the potential to maintain high adherence and effectively train upper-limb function. In addition, these aspects may help to make HBR more goal-oriented, potentially enhancing stroke survivors’ awareness of arm use in daily life.

In line with previous studies, this study highlights the need for HBR systems to have three demands in particular: (1) customization to emphasize the need to tailor HBR technology to individual patients [10,19,25-27]; (2) motivational elements, including features such as reminders [19], feedback [10,12,18,19,25-28], a variety of games or levels [7,10,12,19,26,29,30], and the ease of use [7,10,19]; and (3) active engagement of the therapist to ensure proper execution, progress monitoring, and provision of feedback [7,10,19,25,28-30]. These general recommendations for HBR should apply not only during exercises but also in daily life. HBR should be customized to support daily life goals, motivate patients to use the affected upper limb in daily activities, and provide insights into daily-life patterns that are useful for therapists. Since the primary goal of upper-limb rehabilitation is to promote active use of the affected limb in everyday life, these requirements and factors help facilitate the transfer of rehabilitation gains to daily life.

Several studies have underscored the importance of active engagement of the therapist during HBR (ie, active monitoring) to enhance patient adherence and progress [7,10,28-30]. Additionally, HBR can highlight specific goals and therefore provide useful insights for therapy [31]. This study investigated the presence of a virtual coach in detail, highlighting the importance of its interaction with the patient. It can help the patient with daily structure by using reminders and feedback [32]. On a more detailed level, this study found that measuring both the quantity and quality of movements during daily life and exercises and providing goal-based feedback are crucial, as this may help to control compensatory movements and provide input for therapists to further tailor rehabilitation. Only two studies investigated user requirements for home monitoring in detail [19,33]. However, Langerak et al [19] investigated home monitoring using sensors and Cavuoto et al [33] used a smartphone, whereas this study focused on the use of a smartwatch. Still, all three studies revealed similar results. This indicates the need for careful consideration of monitoring methods and the type of feedback provided to stroke survivors.

This was also the first study that took the perspectives of various end users on the user requirements of NEST into account. Previous studies mostly focused on stroke survivors or held focus group sessions or interviews with the stroke survivor and caregiver together, indicating the need for insights from therapists and caregivers independently, free from influence of the stroke survivor [10,25]. Studies that have included stroke survivors, caregivers, and health care professionals have generally found minimal differences in user requirements between these groups [7,34]. However, despite the significant overlap in user requirements among end users, some minor differences were noted. This suggests the need to pay attention to these differences when further developing the NEST system, as tailoring the systems to accommodate these nuances could enhance its effectiveness and user satisfaction. Caregivers did not express opinions on the early introduction of the system, quality measurement, or goal-oriented feedback. Instead, they emphasized the importance of maintaining their role as a partner rather than a therapist. The absence of these therapeutic considerations from the caregivers’ answers suggests a more social perspective of their role, most likely because they are not directly involved in the therapy process. Consequently, caregivers may view themselves primarily as supporters rather than as system users. Previous reviews have showed that social support positively influences both the quality of life and physical recovery of the stroke survivor, underscoring the need to consider caregivers in HBR [35,36]. Interestingly, the user requirements mentioned by stroke survivors were also mentioned by therapists and caregivers, suggesting that these groups have a good understanding of the stroke survivors’ needs.

In line with the high overlap between end users, there was a strong consensus on user requirements across countries. A previous review showed an agreement across 15 countries on the importance of place, relationships, and therapy [25], though it only focused on HBR without incorporating technology. In addition, a study comparing HBR with technology between Bangladesh and the United Kingdom found similar user requirements but noted more sociotechnical challenges in Bangladesh [37]. In this study, only subtle differences in addressed topics were noted, including privacy concerns raised at the Spanish site, negative caregiver influence at the Dutch site, and self-awareness at the Italian site. The minor differences may reflect the inclusion of sites exclusively from Western countries, where major cultural variation is less likely. Although only small, these differences should be taken into account when further developing the NEST system. Specific social aspects, such as social stigma and relation dynamics among users, were not explicitly addressed nor raised by end users during the focus groups in any country. However, these topics might be more country-specific and could contribute to national differences. The variations in this study may stem from the distinct participant characteristics between the countries. Dutch participants in the chronic phase of stroke recovery likely offered more comprehensive insights into experiences with caregivers at home, unlike the acute stroke survivors at the Italian and Spanish sites. The literature supports that stroke over time affects the experience of being at home, for example, a change of feeling in bodily routines and relationships [38]. In addition, the high motivation observed among Italian stroke survivors may have highlighted the self-awareness. These findings indicate that clinical characteristics, home environments, and coping mechanisms influence the perceptions of HBR by stroke survivors.

One limitation of this study was the predominance of male stroke survivors and the age imbalance among participants across different clinical sites. This could have influenced the needs of the stroke survivors for NEST, since age and sex can influence the severity of stroke [39,40]. Also, women more often live alone after hospital discharge [39]. Additionally, the inclusion criteria for this study, including minimal cognitive impairments, no aphasia, and smartphone usage, likely do not represent the entire stroke survivor population. These inclusion criteria may have contributed to a more positive attitude toward NEST. However, despite the imbalance, our results are aligned with those of previous research, and there was a high degree of overlap in findings across all countries. Another limitation was the participants’ difficulty in conceptualizing NEST, which was still in development. To address this, videos of the RGS app were shown, and when participants asked for more clarity, they were asked to describe their ideal scenario. This approach often led to open discussions about potential features and improvements. The study also faced limitations due to the use of varied interview formats (focus groups, online focus groups, and interviews) imposed by the constraints of the COVID-19 pandemic. Interviews may generate a broader range of items, potentially enhancing depth, whereas focus groups may elicit more personal information due to a peer environment [41]. Furthermore, the online focus groups may have had constrained discussion due to the less natural setting. Nevertheless, previous research showed that content generated in either interviews or focus groups have a high overlap [41], and the same applies to physical or online focus groups [42-44]. Furthermore, the use of identical interview guides ensured consistency in discussion topics. The high degree of overlap in results suggests that the same questions were effectively addressed across the countries. Therefore, this aspect was not considered further in the data analysis. Lastly, no specific questions on between-country or end user differences, such as culture or care pathways, were included, as the focus remained on the primary aim, including user requirements for system development. These topics could have highlighted potential differences; yet, they were not raised by participants, suggesting limited perceived relevance. Therefore, more research is needed on this topic.

In conclusion, all end users across the participating countries emphasized the importance of integrating gamified exercises, monitoring, and a virtual coaching component into an HBR system. The user requirements for such a system can be categorized into three key areas, customization, motivational elements (including ease of use), and feedback elements, which are essential for maximizing user engagement and effectiveness. These areas extend beyond HBR exercises and are also important for daily-life activities. Users’ needs found in this study are largely similar; however, subtle differences between end users (eg, social perspective caregivers) and western countries (caregiver burden, privacy concerns, and self-awareness) should be considered when further developing the NEST system.

## Supplementary material

10.2196/79382 Multimedia Appendix 1 Interview guide used during the focus group sessions and interviews.

## Abbreviations

AWA: agent for well-being assistance
HBR: home-based rehabilitation
NEST: Neurorehabilitation Ecosystem for Sustained Therapy
RGS: Rehabilitation Gaming System
SM: Sint Maartenskliniek
VH: Vall d’Hebron University Hospital
