# Supplementary material for: User Requirements of the Integrated Home-Based Rehabilitation Tool, Neurorehabilitation Ecosystem for Sustained Therapy: Multicenter Focus Group Study With Stroke Survivors, Caregivers, and Health Care Professionals
Source: JMIR Rehabil Assist Technol. 2026 Apr 29;13:e79382. doi: 10.2196/79382 (PMC13153746; doi:10.2196/79382)
Supplement: Multimedia Appendix 1 [file rehab-v13-e79382-s001.pdf]

Multimedia appendix 1: Interview guide

**Introduction**
**Goal**<br>- The overall goal of this project is to develop an application to use at home. This application will help patients who had a stroke to improve their arm–hand function. This is a home–based rehabilitation program, therefore the therapist will not be present during the exercises. We will show you a movie where the application will be explained. → **show movie**<br>- Thus, the app consists of three different parts:<br>1. the RGS–mobile app<br>o The app consists of daily exercises. The exercises are several arm movements. This app also contains games in which you have to move the phone through the environment, while holding it in your hand.<br>o There are also games to play on the phone itself, these focus on cognitive training.<br>2. The second part is the RGS–wear, this is a smartwatch that tracks the activity of the affected arm during the day. This smartwatch can measure how much arm movements you make in one day. This can then be communicated with the therapist.<br>3. The last part is the AWA–coach (agent for wellbeing assistance), this is an app for the smartwatch that will motivate you to use your arm and hand more by doing exercises. The coach will also give feedback about how much you use your arm.<br><br>During this talk, we will discuss the app, the smartwatch and the AWA–coach one by one.
**Home rehabilitation – questions**
**Do you like the idea of home rehabilitation in general?**<br>**When defined as independently (without a therapist), with the help of a technical device (such as an app, computer program or sensors to monitor leg or arm movement)**<br><br>- What do you need to be able to use home–based rehabilitation in daily practice?<br>- Would you prefer to have more home–based rehabilitation or more on site rehabilitation? What should be the ratio between these? When do both need to be implemented? (straight away, after 3 months etc.)<br>- How would you envision having contact with the therapist while being in a home–based rehabilitation project?<br>- What should be the function of therapists and caregivers in home–based rehabilitation?
**RGS–mobile app – questions**
**What do you need in order to be able to use the RGS–mobile app in daily practice?**<br><br>- Would you like to use this app? Why / why not?<br>- Do you think you have the capabilities to use the app independently? In terms of technical skills, arm function for controlling the app etc. Also in terms of description / manual / explanation of the app<br>- What do you think of the different exercises and games?<br>- Which of these parts do you like most?

- Do you think the app would have extra value if you were able to contact your therapist with the app?
- Would you like to play these games with fellow stroke people? Would you like to play with or against each other?
- Would you like to play these games with family or friends? What about being able to share your results?

What would be barriers for you to use the RGS–mobile app in daily practice?

- Do you think you would have enough motivation to use this app?
- Do you think you will experience technical problems?
- Do you have concerns about privacy?
- Which part of the app would you use less?

RGS–wear

What do you think about the daily use of this smartwatch?

- Would you wear the smartwatch all day? Why / why not?
- Recharging
- Able to read and understand the smartwatch
- Aesthetic role (ugly or not)
- Would you like to take it off sometimes? For example during exercising, showering, gardening? What if you *have* to take it off at those times?
- Do you think the smartwatch has an added value for NEST?
- Would you prefer an apple or android smartwatch?
- Do you want your data to be sent to the therapist? Would you prefer this to happen automatically or to be able to decide yourself what data will be sent? What if it will be uploaded to your electronic patient file (only appliable when the hospital works with this)?

AWA–coach – questions

What way would be most suitable for you to get motivated to do exercises?

- What do you think of getting motivated to start doing exercises?
- What do you think of receiving feedback during exercises about how you are doing?
- What do you think of receiving feedback about arm activity after a period of time? How long should this period be?
- What kind of feedback would you like to receive from your therapist? How often should this feedback be given?
- Would you like to give yourself feedback after an exercise or game? In what way would you like to do this (rating, smileys etc.)
- What way would be best for you to receive feedback? A voice, a message, a beep, a vibration of the smartwatch?
- Would you like it if you can make a personal avatar as a coach? For example, by adding a picture of your grandchild while the coach gives feedback?
- Would you like to receive reminders to start playing games or do exercises?
- We talked about different forms of feedback, could you indicate which feedback is most important and which the least?

NEST – questions (20 min)
**What do you think about the concept of NEST?**<br>- Would you like to use NEST?<br>- What are demands to be able to use NEST?<br>- What kind of problems do you anticipate when using NEST?<br>- What do you think is the biggest advantage of NEST?<br>- Do you have enough technical skills to use NEST on a daily basis?<br>- Would you pay for NEST? If yes, how much?<br>- How often or how long would you like to use NEST?<br>- Would you recommend NEST to others?<br>- Would you like to get in touch with other people who had a stroke or to be able to have rehabilitation together?
**Closing**
- Give a summary of the interview and ask if this is correct

The interview guides for healthcare professionals and caregivers included the same questions as the guide above, with adaptations to align with their perspectives when addressing stroke survivor–specific topics. For example:

- Stroke survivor: Do you think you have the capabilities to use the app independently?
- Therapist: Do you think the patient has the capabilities to use the app independently?
- Caregiver: Do you think your loved–one has the capabilities to use the app independently?

Additional questions were included for both caregivers and therapists to capture their personal perspectives. Extra questions for the **therapist:**

- RGS app
  - Would you like it if patients could contact you through the app?
  - Do you think you have the time or want to make time to get to know this app and explain it to patients?
- Wear
  - Do you think the smartwatch has an added value for NEST?
  - Would you use the data that the smartwatch produces? (i.e., meetings with patient)
- Coach
  - Would you like to give feedback to the patient based on the data of the smartwatch? How often would you like to do this? What kind of feedback would you give?

For the **Caregivers:**

- General HBR
  - What would be advantages and disadvantages of home rehabilitation for you?
- App
  - Would you be willing to help your loved–one with using the app?
- Wear
  - Would you appreciate if you have insight in the activity measured with the smartwatch?
  - Would you be willing to help your loved one with using the smartwatch?
